# Cardiac tamponade and paroxysmal third-degree atrioventricular block revealing a primary cardiac non-Hodgkin large B-cell lymphoma of the right ventricle: a case report

**DOI:** 10.1186/1752-1947-5-433

**Published:** 2011-09-05

**Authors:** Zied Frikha, Leila Abid, Dorra Abid, Souad Mallek, Imed Frikha, Mohamed Abdennadher, Noomen Rekik, Samir Kammoun

**Affiliations:** 1 Cardiology Department, Hédi Chaker Hospital and Sfax Medical University, 3029 Sfax, Tunisia; 2 Cardiovascular Surgery, Habib Bourguiba Hospital, 3029 Sfax, Tunisia

## Abstract

**Introduction:**

Primary cardiac lymphoma is rare.

**Case Presentation:**

We report the case of a 64-year-old non-immunodeficient Caucasian man, with cardiac tamponade and paroxysmal third-degree atrioventricular block. Echocardiography revealed the presence of a large pericardial effusion with signs of tamponade and a right ventricular mass was suspected. Scanner investigations clarified the sites, extension and anatomic details of myocardial and pericardial infiltration. Surgical resection was performed due to the rapid impairment of his cardiac function. Analysis of the pericardial fluid and histology confirmed the diagnosis of non-Hodgkin large B-cell lymphoma. He was treated with chemotherapy.

**Conclusion:**

The prognosis remains poor for this type of tumor due to delays in diagnosis and the importance of the site of disease.

## Introduction

Primary cardiac tumors are rare. Cardiac lymphoma is the rarest primary cardiac tumor and it is usually fatal. The prognosis is poor because of diagnostic delay and the importance of the site of disease. It often begins with a pericardial effusion. Its treatment is based on chemotherapy.

## Case presentation

**Figure 1:**
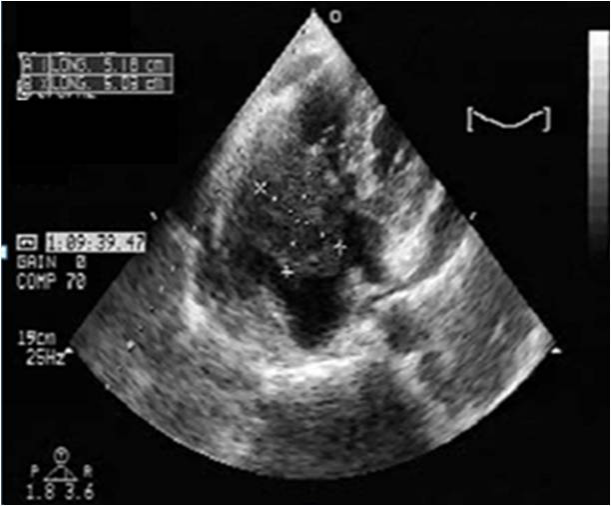
**Transthoracic echocardiography four chamber view showing a pericardial effusion and a large mass**. The mass measured 5.5 cm× 5 cm in the right ventricle and was attached to the tricuspid valve creating a tricuspid stenosis. The tumor has spread over the right atrium.

**Figure 2:**
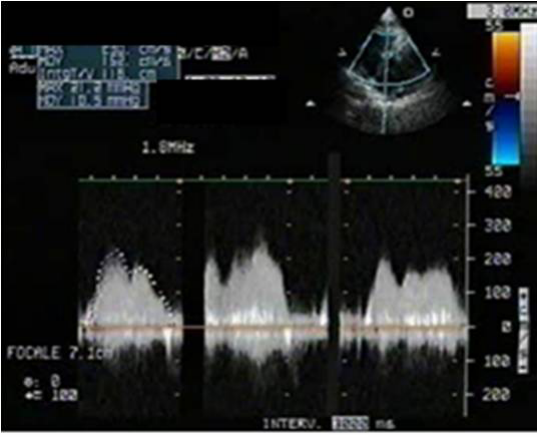
**Continuous wave Doppler**. The tumor created a hemodynamic tricuspid stenosis which is a sign of high right ventricle inflow velocities.

**Figure 3:**
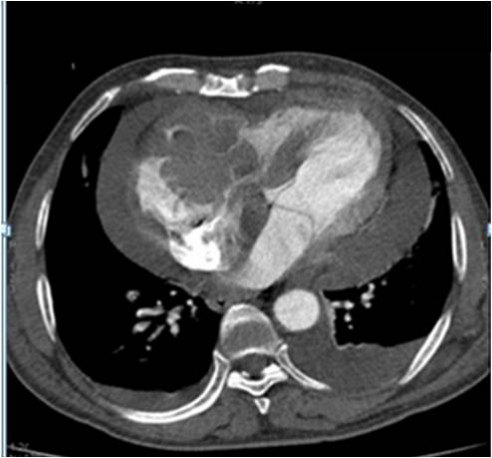
**Coronary angiography showing an accentuation of myocardial blush**.

**Figure 4:**
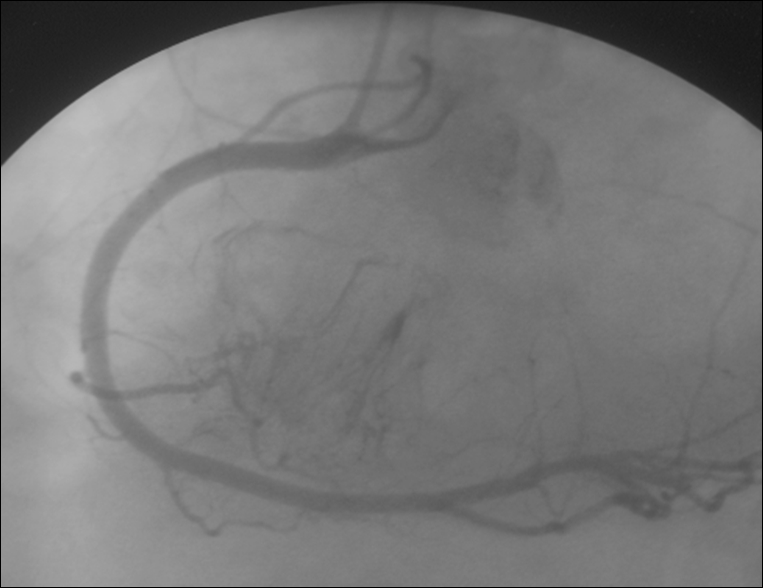
**Computed tomography scan showing the presence of a right heart tumor developing on both sides of the tricuspid valve**.

A 64-year-old immunocompetent Caucasian man with no history of cardiac disease presented with chest pain, dyspnea and edema of his lower limbs associated with a degeneration of his general state. On physical examination he had a temperature of 37°C, blood pressure of 100/74 mmHg, and heart rate of 30 bpm. His jugular venous pressure was high. The first and second heart sounds were normal without any audible murmurs, rubs or gallops. His chest was clear to auscultation. His hemogram, hepatic enzymes and inflammation markers were all normal. The patient was HIV-negative. His chest X-ray revealed cardiomegaly as well as bilateral pleural effusion. The standard 12-lead ECG indicated an atrioventricular block of the third-degree. It returned to normal spontaneously one hour later. Transthoracic echocardiography (TTE) (Figures 1 and 2), demonstrated not only a pericardial effusion of 23 mm by 35 mm with signs of tamponade but also the presence of a large mass at the level of the right ventricle. The mass had a wide base and was heterogeneous. It appeared lobulated with a tissular echo texture that measured 5.5 cm by 5 cm. It was also attached to the tricuspid valve creating a right ventricle inflow obstruction. The tumor spread over the right atrium. He underwent an urgent pericardial drainage which returned 600 cm^3 ^of hemorrhagic liquid. Bacteriological and cytological analyses revealed large cells suggestive of a lymphoproliferative disorder. A computed tomography scan showed the presence of a right heart tumor on both sides of the tricuspid valve as well as peritoneal effusion. No other organ involvement was observed (Figure 3). Coronary angiography accentuated an increase of a myocardial blush in favor of the highly vascular nature of the tumor (Figure 4). This examination was performed because the patient was more than 40 years old. It was thought that emergency surgery might be necessary at any time because of size of his tumor.

Due to the rapid impairment of his cardiac function and the life-threatening hemodynamic instability, an echocardiography was performed which showed an obstruction of the right ventricle inflow.

He underwent an emergency thoracotomy. The purpose of this surgery was not to remove the entire tumor. It was limited to freeing the tricuspid valve and the intra-right ventricle obstruction. Surgical resection of the mass was difficult and incomplete. The tumor had infiltrated his right atrium, the atrioventricular septum and the proximal side of the right ventricle. Surgical removal was laborious but without complications.

**Figure 5:**
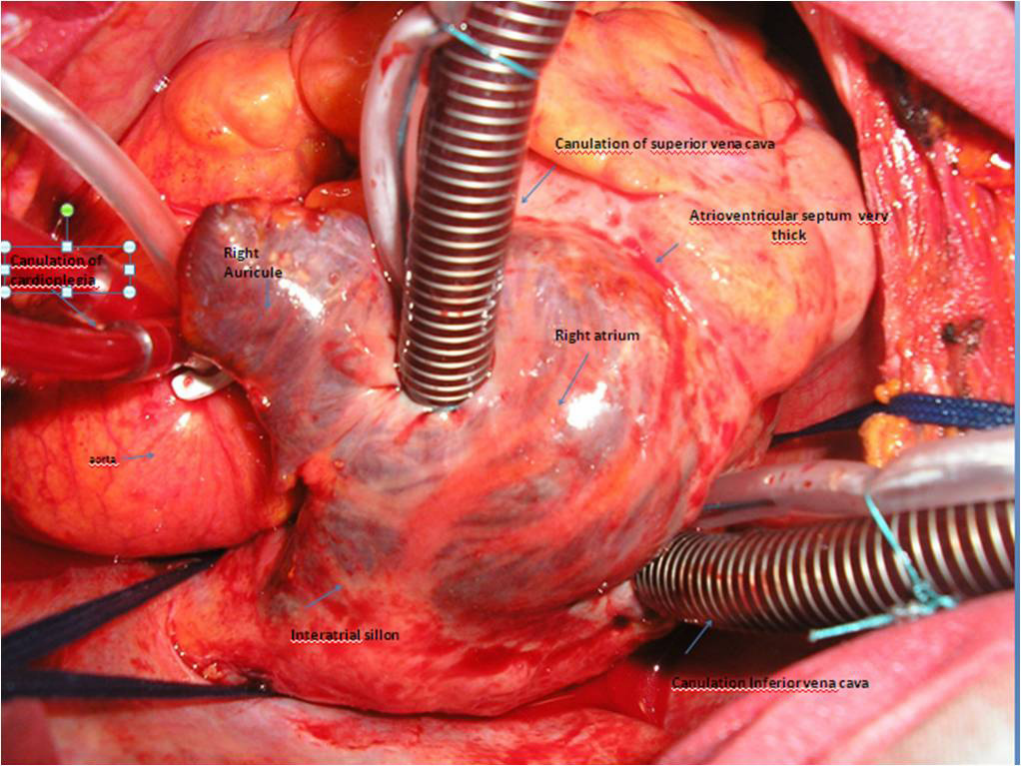
**Macroscopic aspect of the tumor**. The tumor is infiltrating the right atrium, the atrioventricular septum and the proximal side of the right ventricle.

**Figure 6:**
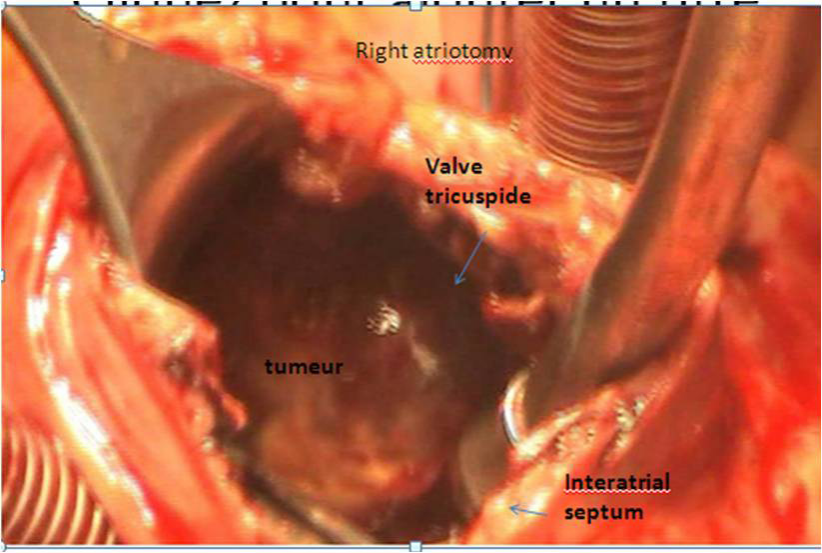
**Macroscopic aspect of the tumor which is infiltrating the right atrium**.

The tumor was submitted to the pathology laboratory as white and red soft fragments measuring 3 cm by 3 cm by 2 cm (Figures 5 and 6). Histological analysis revealed non-Hodgkin large B-cell lymphoma (CD45+ CD20+ CD3- BCl2+ CD20- CD10- BCl6-). The lymphoma was classified as IE, according to the Ann Arbor staging classification.

Chemotherapy with the R-CHOP (Rituximab, Cytoxan, Hydroxydaunorubicin (Adriamycin), Oncovin (Vincristine), Prednisone/Prednisolone) regimen began immediately after resection.

**Figure 7:**
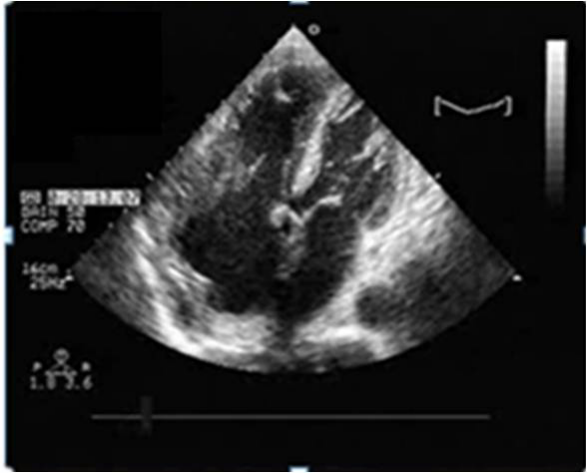
**A transthoracic echocardiography four chamber view showing a reduction of the size of the mass**.

After the first course of chemotherapy TTE demonstrated a reduction in the size of the mass (Figure 7).

## Discussion

Primary cardiac tumors are extremely rare in immunocompetent persons. They are more frequent in patients with acquired immunodeficiency syndrome (AIDS) or in transplant recipients. This was not the case in our patient. Approximately 25% of primary cardiac tumors are malignant. Cardiac tumors are classified according to their location and the degree of intra-cavitary obstruction. It is interesting to separate primary cardiac lymphoma in which cardiac events are the first indications, from secondary locations in which general events are predominant and the discovery of the cardiac involvement is often fortuitous [1].

Primary cardiac lymphoma is an extranodal non-Hodgkin lymphoma exclusively located in the heart and/or pericardium [2]. It represents 1.3% of primary cardiac tumors (PCL) and less than 1% of all lymphomas [2-4]. The right atrium and right ventricle are the two most frequently involved sites with two-thirds of cases involving the right atrium [2-5].

Clinical presentations associated with primary cardiac lymphoma are heterogeneous. They are generally related to the site of involvement in the heart which makes early diagnosis difficult. In their series, Fuzellier *et al*. reported right-sided heart failure, dyspnea, tamponade and arrhythmias as the most frequent manifestations [2]. Cardiac tamponade is a frequent mode of presentation. The association of a tamponade with an alteration of the general state or the general signs leads directly to a neoplasia disease [2-6]. Congestive heart failure is explained by myocardial involvement. The disorders of conduction are the consequence of the invasion of the inter-atrial septum likely with an extension to the nodal tissue. The mechanism of cardiac arrhythmia could be the infiltration of the roof of the side wall of the right atrium by the tumor tissue [7].

TTE visualizes the pericardial effusions easily. It also allows an estimation of its tolerance and reveals the presence of any intracardiac mass. TEE is considered an initial imaging method when an intra-cardiac mass is doubtful [8,9]. *'*It is better for identifying tumoral masses, allowing suspicion for an infiltrated cardiac tumoral mass to be a primary cardiac lymphoma' [6]. The sensitivity of TEE for the detection of primary cardiac lymphoma approaches 100% in some series in specialized units that have experience with this kind of laboratory investigation [5]. It is also a good follow-up examination allowing the verification of the regression of the tumor after chemotherapy in a few centers [5]. TEE is excellent at visualizing tumors in the atria, but much less so for anterior masses (for example, near the right ventricular apex), where TTE is superior. In our department, we are experienced in diagnosing cardiac tumors and monitoring their regression by TTE. Computed tomography allows the delineation of the cardiac mass and the specification of its connections with the cardiac structures as well as the extent of the disease. An MRI becomes the examination reference for the diagnosis of cardiac tumors. It offers superior anatomic details of myocardial and pericardial infiltration. This examination can also serve as a reference for the follow-up of patients undergoing chemotherapy [2]. However, fast moving tumors (such as some myxomas) will adversely affect the quality of the MRI image. In our patient who had an auriculoventricular block of the third degree in the electrocardiogram, echocardiography followed by computed tomography can help in arriving at a hypothesis to explain the origin of the conduction disorder. It is probable that the tumor in this case report invaded the inter-atrial septum and the atrioventricular node. We do not have any explanation for why it was paroxysmal. It is probable that the inflammation process around the tumor is the cause. Cytological analysis of the pericardial liquid does not always permit a diagnosis because the effusion can be reactive [2]. The cytology results in the pericardial fluid are often nonspecific. It demonstrates atypical lymphoid cells [5]. Most cases require biopsy or surgical excision for diagnosis [2]. In the presence of a right-sided cardiac mass, an aggressive approach to obtain a rapid histological diagnosis is important. Less invasive procedures, such as TEE guided biopsy, endomyocardial transvenous biopsy, mediastinoscopy and thoracoscopic pericardial window have been performed with success [5].

The treatment of primary cardiac lymphoma is not clearly codified. It differs according to the clinical team. Surgical treatment is discouraging because surgical resection of primary cardiac lymphoma is often difficult and incomplete. It is reserved for patients with life-threatening hemodynamic compromise caused by mechanical complications (as was the case with our patient) or tamponade [7]. Early systemic treatment appears to be the only chance for cure.

Chemotherapy remains the preferred initial treatment. It should be guided by the immunohistological characteristics of the lymphoma and its extension to other organs. At the end of the treatment, we can expect a reduction of any rhythm disorders due to regression of the tumor mass [6].

## Conclusion

Primary cardiac lymphoma is rare. The presence of a right cardiac mass raises the possibility of primary cardiac lymphoma. Echocardiography is the preferred procedure for diagnosis and follow-up. In addition, it allows an estimation of the hemodynamic state. Rapid histological diagnosis is important because systemic therapy can influence the prognosis in the presence of a primary cardiac lymphoma [2].

## Consent

Written informed consent was obtained from the patient for publication of this case report and accompanying images. A copy of the written consent is available for review by the Editor-in-Chief of this journal.

## Competing interests

The authors declare that they have no competing interests.

## Authors' contributions

ZF, LA, DA, SM, and SK analyzed and interpreted the patient data and treat it. IF performed the surgery. All authors were major contributors in writing the manuscript. All authors read and approved the final manuscript.

## Acknowledgements

We thank Pr Mourad Hentati for his collaboration to elaborate this observation and for the care that he provided for the patient.
